# Effects of Web-Based Physical–Cognitive Telerehabilitation Exergaming on Physical and Cognitive Performance in Chronic Stroke Survivors: Protocol for a Randomized Controlled Trial

**DOI:** 10.3390/jcm15082945

**Published:** 2026-04-13

**Authors:** Puntarik Keawtep, Sirinun Boripuntakul, Piangkwan Sa-nguanmoo, Pranglada Jearjaroen, Cattaleeya Sittichoke, Teerawat Kamnardsiri, Thepparit Sinthamrongruk, Somporn Sungkarat

**Affiliations:** 1 Integrated Neuro-Musculoskeletal, Chronic Disease, and Aging Research Engagement Center (ICARE Center), Department of Physical Therapy, Faculty of Associated Medical Sciences, Chiang Mai University, Chiang Mai 50200, Thailand; puntarik.k@cmu.ac.th (P.K.); sirinun.b@cmu.ac.th (S.B.); piangkwan.s@cmu.ac.th (P.S.-n.); pranglada.j@cmu.ac.th (P.J.); cattaleeya.sit@cmu.ac.th (C.S.); 2 Department of Digital Game, College of Arts, Media, and Technology, Chiang Mai University, Chiang Mai 50200, Thailand; teerawat.k@cmu.ac.th; 3 College of Arts, Media, and Technology, Chiang Mai University, Chiang Mai 50200, Thailand; thepparit.s@cmu.ac.th

**Keywords:** stroke, cardiovascular disease, exercise, telerehabilitation, cognitive function, health, study protocol

## Abstract

As stroke survivors often manifest not only physical dysfunction but also cognitive impairment, interventions that address both physical and cognitive aspects may be beneficial for individuals with chronic stroke. However, a randomized clinical trial (RCT) examining the effects of physical–cognitive telerehabilitation exergaming across physical and cognitive domains in chronic stroke survivors remains scarce. Therefore, this study aims to investigate the effectiveness of the web-based physical–cognitive telerehabilitation exergaming on the physical and cognitive performance of chronic stroke survivors as compared to control. A two-arm RCT with a blinded assessor will be conducted. Fifty-two participants will be randomly assigned to either the intervention or the control group. Participants in the intervention group will receive a 12-week training program, whereas those in the control group will continue with usual care. Motor function, gait speed, balance, fall risk, muscle power, weight distribution, body composition, global cognition, executive function, attention, memory, as well as enjoyment will be assessed. Existing evidence has established the health benefits of physical–cognitive exergaming. It is expected that a physical–cognitive exergaming program may further exhibit positive effects on both physical function and cognition beyond the results of usual care. In addition, incorporating physical–cognitive training into a web-based telerehabilitation program may improve adherence and provide a feasible and enjoyable rehabilitation option for individuals with chronic stroke.

## 1. Introduction

Stroke survivors commonly experience muscle weakness, impaired balance, and reduced mobility, which substantially limit activities of daily living, community participation, and functional independence [1]. In parallel with physical dysfunction, cognitive impairments are frequently reported after stroke and contribute to long-term disability [1,2,3]. Previous studies have shown that cognitive impairment affects 27% of stroke survivors at 3 months and 21% at 12 months after stroke and is associated with poorer quality of life and activities of daily living at 1 year [2]. A meta-analysis of 27 studies demonstrated that poststroke cognitive impairment is associated with a 59% increased risk of recurrent stroke and a twofold higher risk of mortality [3]. Therefore, preventing poststroke cognitive impairment among at-risk individuals and promoting health throughout stroke rehabilitation are critical for reducing stroke-related disability.

It has been well documented that physical exercise and physical activity are recommended as an effective non-pharmacological intervention to improve or restore physical function after stroke [4,5]. The therapeutic approach to stroke rehabilitation requires repetitive training combined with continuous modification of training programs to maintain patient engagement and optimize long-term functional outcomes [6]. However, a previous systematic review reported that common perceived barriers to engaging in or continuing physical activity after stroke include lack of motivation, limited access to rehabilitation services, transportation difficulties, and cost, as well as health concerns and stroke-related impairments [7]. In addition, adherence to physical interventions among stroke survivors has been shown to decrease by 30–50% within the first year [8]. To mitigate these barriers, an intervention that supports care delivery and incorporates accessible, safe, and enjoyable elements may facilitate greater physical activity and adherence to regular exercise among stroke survivors. Digital health interventions, including exergaming and telerehabilitation, have been integrated into healthcare, providing accessible and motivating interventions, extending services to underserved areas, and reducing costs and travel burden [9,10]. Research evidence has demonstrated that exergaming has positive effects on balance and functional mobility in chronic stroke survivors [11]. Allegue et al. [12] demonstrated that exergame-based telerehabilitation improved motor function and movement quality after 8 weeks of training. Ballester et al. [13] reported that remotely delivered exergame-based training for upper-limb rehabilitation improved the functional ability of the paretic arm and hand, enhanced maximal finger flexion, and increased neuroplastic changes in stroke survivors. Burgos et al. [14] showed that a digital health intervention incorporating inertial movement sensors and a web-based exergame platform for remote balance training by a physiotherapist improved balance and activities of daily living among stroke survivors after 4 weeks of intervention. In addition to improving physical health, the interactive features of exergaming and gamification enhanced motivation, leading to greater adherence compared with regular exercise and providing an enjoyable and effective approach for stroke patients [15,16,17].

Research evidence has demonstrated that physical–cognitive training benefits not only physical or motor function [18,19,20] but also cognitive and brain health [21,22,23,24]. Previous studies demonstrated that physical and cognitive training improved the cognitive functions and independent living ability of older adults with and without cognitive impairment [21], enhanced executive function in patients with early-stage Alzheimer’s disease [24], and improved balance and gait and reduced fear of falling and fall incidence in patients with cerebrovascular disease [18]. Among stroke survivors, existing evidence suggests that physical and cognitive training is more effective than single-modality interventions or usual care in improving cognitive and functional outcomes after stroke [25,26,27]. A previous randomized controlled trial (RCT) demonstrated that physical and cognitive training improved cognitive function, with cognitive benefits maintained at the 6-month follow-up in stroke survivors [27]. A systematic review and meta-analysis study indicated that physical–cognitive training improved the cognitive function of poststroke cognitive impairment, particularly in the domain of executive function [28]. According to cognitive–motor interference theory, the combination of motor and cognitive tasks places demands on attentional capacity and the integrity of executive functions [29]. A previous study reported that combined motor and cognitive tasks increased task complexity, enhanced brain activation and functional connectivity, and facilitated the engagement of distributed neural networks [30]. Collectively, physical–cognitive interventions may yield synergistic effects and lead to superior outcomes compared with usual care or single-modality training.

To the best of our knowledge, no previous studies have investigated the effects of physical–cognitive training on both physical and cognitive outcomes in chronic stroke survivors, particularly across a comprehensive range of domains, including motor function, gait speed, balance and limits of stability, fall risk, muscle power, and weight distribution, as well as global cognitive function, executive function, memory, and attention. Additionally, there is a limited number of studies that have incorporated physical–cognitive training with web-based exergaming and a gamification approach into telerehabilitation to promote engagement and adherence to exercise. Therefore, this study aims to investigate the effectiveness of the web-based physical–cognitive telerehabilitation exergaming on the physical and cognitive performance of chronic stroke survivors as compared to control. Given the health benefits of combined training and the accessibility of digital health interventions, it is hypothesized that web-based physical–cognitive telerehabilitation exergaming would be beneficial to chronic stroke survivors and may potentially provide greater physical and cognitive benefits than control conditions.

## 2. Materials and Methods

### 2.1. Aims

The purpose of this study is to investigate the effectiveness of the web-based physical–cognitive telerehabilitation exergaming on the physical function and cognitive performance of chronic stroke survivors as compared to the control condition.

### 2.2. Study Design

**Figure 1:**
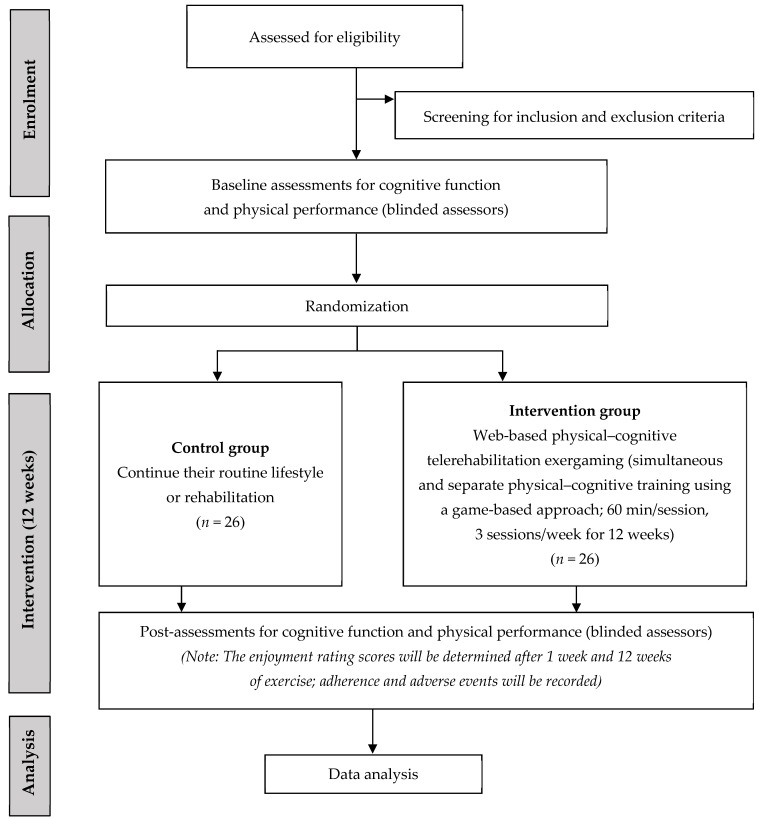
Flow chart of the study procedure.

This study is designed as a two-arm, assessor-blinded randomized controlled trial. The study protocol was developed in accordance with the Consolidated Standards of Reporting Trials (CONSORT) guidelines, and the study flow diagram is presented in Figure 1. The SPIRIT 2025 checklist is provided in Table S1 (Supplementary Materials). The study protocol was registered at ClinicalTrials.gov (NCT07446062). Recruitment began in February 2026 and is estimated to be completed in March 2027.

### 2.3. Settings and Participants

The intervention will be delivered in a home-based format at each participant’s home. Participants will be included if they have been diagnosed with ischemic or hemorrhagic stroke at least 6 months before enrolment [31], are aged between 20 and 80 years [31], are able to walk at least 10 m with or without assistive devices [32], are able to perform sit-to-stand safely with or without assistive devices, can see the screen of a tablet or notebook computer at a distance of at least 60 cm [33], have a stable medical condition and are able to follow study instructions [32], and are frequent users of a mobile phone, tablet, or notebook computer. Participants will be excluded from the study if they have medical conditions or complications that would be unsafe to exercise [31], have neglect, impaired communication, or uncorrected visual problems [32], or have severe spasticity of the upper or lower extremities (Modified Ashworth Scale ≥ 3) [31,32].

### 2.4. Participant Characteristics

Participant characteristics including age, body mass index, gender, and education level will be recorded. Medical information regarding stroke characteristics, including stroke type, paretic side, and time since stroke onset will also be collected.

### 2.5. Sample Size

The sample size was calculated using G*Power version 3.1.9.2 based on outcome measures. Effect sizes were derived from previous studies, which reported values of 0.46 for executive function [34], 0.57 for fall risk [35], and 0.37 for gait speed [36,37]. With a power of 0.80 and an alpha level of 0.05, sample sizes of 30, 20, and 46 will be required to detect a change in executive function, fall risk, and gait speed respectively. Accordingly, a total sample size of 46 participants is considered sufficient to detect changes in outcome measures. To accommodate a 10 percent dropout rate, a total of 52 participants (26 per group) will be enrolled in the study. However, the appropriate sample size will be recalculated using data from our preliminary study.

### 2.6. Randomization and Allocation Concealment

Eligible participants will be randomly allocated to the intervention or control group in a 1:1 ratio. Randomization will be performed by an independent researcher using a computer-generated randomization sequence created with SPSS version 25. Allocation concealment will be ensured using sealed, opaque, and sequentially numbered envelopes, which will be opened only after baseline assessment and participant enrolment. All outcome assessments will be conducted by trained assessors who are blinded to group allocation. Due to the nature of the intervention, neither the physiotherapist delivering the intervention nor the participants can be blinded.

### 2.7. Procedure

Participants will be recruited through both online and offline advertisements within local communities. Written informed consent will be obtained from eligible participants before participation in the study. All participants will be assessed for physical function (motor function, gait speed, balance and limits of stability, fall risk, muscle power, and weight distribution) and cognitive performance (global cognitive function, executive function, memory, and attention). Physical and cognitive performances will be assessed at baseline and after the end of 12 weeks of intervention. The enjoyment rating scores will be determined after 1 week and 12 weeks of training. A 12-week home-based training program will be implemented in this study. Participants will be encouraged to contact the research team whenever they have questions or concerns.

### 2.8. Interventions

#### 2.8.1. Web-Based Physical–Cognitive Telerehabilitation Exergaming

**Figure 2:**
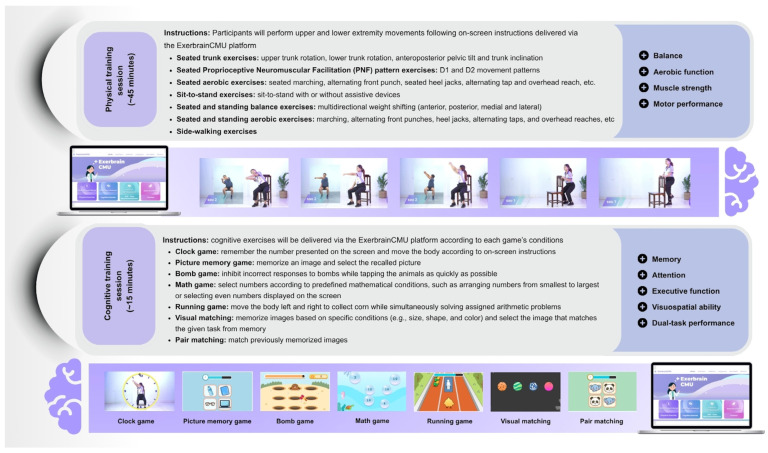
Summary of the characteristics of web-based physical–cognitive telerehabilitation exergaming.

Participants will perform a physical–cognitive exergame via ExerbrainCMU web-based platform at home setting for 60 min/session, 3 sessions/week for 12 weeks. The program will consist of warm-up, physical–cognitive training, and followed by cool-down. The physical–cognitive exergame will consist of trunk exercises, upper- and lower-extremity exercises, exercises using Proprioceptive Neuromuscular Facilitation (PNF) patterns, balance exercises, aerobic exercises, sit-to-stand training, and side-walking exercises, combined with cognitive training targeting executive function, attention, memory, visuospatial ability, and dual-task performance. In general, physical–cognitive exergames will mostly be performed in a sitting position. Task difficulty will be progressively increased by manipulating movement complexity, speed, and directional demands, while cognitive load will be adjusted by varying cognitive complexity, the number of stimuli, task conditions, attentional demand, and memory load. The intervention will comprise three difficulty levels: easy (weeks 1–4), medium (weeks 5–8), and hard (weeks 9–12). Participants scoring below 80% across all games will be required to repeat the current level until the 80% passing criterion is met before progressing to the next level. Instructional materials, including basic instructions and training videos, will be provided to ensure correct and safe execution of the intervention. A summary of the intervention is presented in Figure 2 and Table S2 (Supplementary Materials).

Telerehabilitation will be conducted using the ExerbrainCMU web-based platform. Participants will engage in exercise training at home or in remote areas via simultaneous video conferencing, accompanied by a physiotherapist. The telerehabilitation system consists of a main monitoring controller for physiotherapists, a network data system, and connected end-users who are stroke patients. The main monitoring controller enables therapists to monitor exercise accuracy and safety at weeks 2, 4, 8, 10, and 12. Family members or caregivers may stand beside participants if there are safety concerns.

Prior to commencing the 12-week home-based intervention, participants will practice the exercises under the supervision of a physiotherapist to ensure proper and safe performance. During training, participants will be instructed to monitor their own fatigue, RPE, and movement performance (e.g., task accuracy, movement quality, and completion of prescribed tasks). The RPE will be self-reported and recorded, with target intensities of 12–14 for Levels 1–2 and 15–17 for Level 3. Participants will be advised to reduce the intensity of the exercise, contact the researcher, or terminate the session according to predefined safety criteria if they experience excessive fatigue (e.g., RPE above the target range), discomfort, dizziness, or difficulty maintaining postural stability. Participants will also receive a booklet to record their training activities and any adverse events.

#### 2.8.2. Control Condition

Participants in the control group will continue their usual lifestyle or receive usual care rehabilitation during the study period, with no additional intervention. They will be encouraged to maintain their usual activities and avoid participation in other structured exercise programs outside the study intervention. The type, frequency, and intensity of concurrent treatments will be recorded and treated as covariates in the statistical analysis to control for potential confounding effects. After the 12-week period, they will be invited to participate in the intervention program according to their preference. Participants will be asked to inform the research team if there are any routine changes or unexpected events (e.g., hospital admission, illness).

### 2.9. Intervention Adherence and Adverse Events

Participants in the intervention program will have their training sessions automatically recorded on the ExerbrainCMU web-based platform immediately after each exercise session, including information on session duration, training completion, and training frequency. These data will be used to monitor adherence and engagement throughout the intervention period. Weekly reminders will be sent via social media applications (e.g., LINE and Facebook) to promote adherence. In addition, research staff will conduct scheduled telemonitoring sessions at weeks 2, 4, 8, 10, and 12, during which information regarding participants’ training will be collected and cross-validated with the platform records.

Participants will be instructed to report any adverse events that occur during the study. They will also be asked about any health problems at each assessment point from baseline to 12 weeks. Participants will be informed that they can contact the research team at any time if they have questions or concerns. All adverse events or harms associated with the intervention will be recorded and reported in the study findings.

### 2.10. Assessment and Outcome Measures

Comprehensive health outcomes, including motor function, fall risk, gait speed, balance, limits of stability, muscle power, weight distribution, body composition, executive function, global cognitive function, memory, attention, and enjoyment, will be assessed by trained assessors blinded to participants’ group allocation. Physical and cognitive parameters will be assessed at baseline and the end of the 12-week exercise, while enjoyment rating scores will be determined after 1 week and 12 weeks of intervention. There will be one assessor for each assessment category (cognition and physical function). The assessment sessions will be divided into two days. For the first day, memory, attention, motor performance, gait speed, and fall risk will be measured. For the second day, body composition, global cognitive function, executive function, balance, limits of stability, muscle power, and weight distribution will be determined. An overview of the schedule of enrolment, interventions, and assessments is summarized in Table 1.

#### 2.10.1. Primary Outcomes

Primary outcomes will be executive function and fall risk. The executive function will be measured using the Trail Making Test (TMT) [38]. This test is widely used to assess executive abilities in stroke survivors [39]. Participants will be instructed to draw lines connecting numbers in numerical order (TMT Part A) and numbers and letters in an alternating ascending sequence (TMT Part B) as quickly and accurately as possible [38]. The score for each part represents the time required to complete the task. Besides the direct scores, the B–A difference score will represent an executive function [38].

Fall risk will be assessed using the Timed Up and Go (TUG) test [40]. The TUG test has been demonstrated to be valid and to identify the risk of falling in community-dwelling older adults as well as in stroke patients [40]. Participants will be instructed to stand up from a chair, walk 3 m at their maximum safe pace, turn around, return to the chair, and sit down. Participants will be allowed to wear their usual shoes and use their assistive devices and/or orthoses if needed. The average time to complete the test in 2 trials will be recorded.

#### 2.10.2. Secondary Outcomes

Secondary outcome measures will include global cognitive function, memory, attention, motor function, gait speed, balance, limits of stability, muscle power, weight distribution, body composition, enjoyment, and adherence. Global cognitive function will be assessed using the Montreal Cognitive Assessment (MoCA) [41]. The MoCA is a reliable tool for assessing global cognition in stroke survivors [42]. The maximum possible score is 30 points. To adjust for educational level, one additional point will be added for participants with ≤12 years of education. Memory and attention will be assessed using the Digit Span Test [43]. Participants will be asked to repeat sequences of digits presented verbally either in the same order (forward) or in reverse order (backward). The number of correctly recalled sequences for each condition will be recorded [43].

Motor function, gait speed, balance and limits of stability, muscle power, and weight distribution will be assessed using the Motor Assessment Scale (MAS) [44], 10-Meter Walk test (10MWT) [45], the Modified Functional Reach Test (MFRT) [46,47], 5 times sit-to-stand test (5TSTS) [48], and the Stance Symmetry Test (SST) [49], respectively. For the MAS test, participants will be asked to perform 8 items of functional tasks: supine to side lying, supine to sitting, balance sitting, sitting to standing, walking, upper arm function, hand movement, and advanced hand activities. Each item is scored on a 7-point scale (0–6; 0 = no function to 6 = normal function), based on a person’s ability to perform specific tasks. Participants will perform each task three times and the best performance will be recorded. For the 10MWT, participants will be instructed to complete a 10 m walk at a self-selected speed with or without walking aids [45]. An acceleration distance of 2 m and a deceleration distance of 2 m will set up at the beginning and the rear end. The time will be recorded by digital stopwatch for the middle 10 m distance. Measurements will be determined twice. The speed will be calculated by dividing the distance by time (m/s). Dynamic balance and limits of stability while sitting will be measured by MFRT [46]. Participants will be sitting on a standard chair with their feet shoulder-width apart, flat on the floor, hips and knees at 90° flexion with the non-paretic side closest to the wall. The participant will be instructed to reach as far as possible with the arm horizontal while maintaining balance. The location of the fifth finger at the beginning and the end of the reach will be recorded. MFRT will consist of three conditions including forward reach while sitting with the unaffected side near the wall, lateral reach to the right while sitting with the back to the wall, and lateral reach to the left while sitting with the back to the wall. Each participant will perform a total of five trials per condition. The first two trials will be used for familiarization, and the mean of the final three trials will be used for analysis [46,47]. As for muscle power, participants will be instructed to perform five sit-to-stand repetitions as quickly as possible. The time required to complete the task will be recorded [48]. Weight distribution will be measured using the SST. This test assesses the distribution of weight between both legs. Participants will be instructed to stand with each foot on a separate weighing scale, with the distance between the feet set at 11% of the participant’s height. They will be asked to stand as still as possible for 30 s. The assessors will record the weight borne by each leg at 25 s. Each participant will complete three trials, and the results will be averaged. The symmetry ratio will be calculated as the average weight bearing through the affected leg divided by the average weight bearing through the non-affected leg [49]. Furthermore, minimum detectable difference (MDD) values for the MFRT, 5TSTS, and SST will be used to aid the interpretation of clinical significance. Body fat percentage and fat mass will be measured using a body fat composition monitor with an accuracy of 0.1 kg and 0.1%, respectively [50,51]. In addition, the Physical Activity Enjoyment Scale (PACES) will be used to assess participants’ enjoyment during the intervention [52]. PACES consists of eight items rated on a 7-point Likert scale from 1 (strongly disagree) to 7 (strongly agree). The overall enjoyment score will be derived from the sum of all item scores. Furthermore, adherence to the intervention will be recorded and calculated as the percentage of attended physical–cognitive training sessions using (n/36) × 100.

### 2.11. Statistical Analyses

Descriptive statistics will be used to summarize the demographic data and baseline characteristics of each group. Data normality will be assessed using the Kolmogorov–Smirnov test. Homogeneity of variances will be evaluated using Levene’s test. Independent-samples *t*-tests and chi-square tests will be used to compare demographic characteristics between the intervention and control groups. The primary analysis will be conducted using a two-way mixed-model ANOVA to examine differences in outcome measures between groups across two assessment time points (baseline and 12 weeks). The Bonferroni correction will be used for the post hoc analysis. In case of baseline imbalances, an analysis of covariance (ANCOVA) will be conducted to compare post-intervention outcome measures between groups, with baseline scores and time since stroke included as covariates to control for potential confounding effects. However, if data are not normally distributed, appropriate nonparametric methods will be considered. The primary comparative analyses will follow the intention-to-treat principle. Missing data will be handled using multiple imputation by chained equations, generating five imputed datasets. The imputation model will include all outcome variables, baseline scores, and relevant covariates. Additionally, a per-protocol analysis will be performed for the primary outcomes to evaluate the intervention effects among participants who attended at least 80% of the sessions. Statistical significance will be set at *p* < 0.05.

### 2.12. Artificial Intelligence Statement

During the preparation of this manuscript, the authors used ChatGPT (GPT-5.3-mini) to assist with language editing to improve clarity and readability. No content generation was performed using artificial intelligence. The authors carefully reviewed and edited the content and take full responsibility for the final version of the manuscript.

## 3. Discussion

The present work describes the study protocol of an RCT designed to evaluate the effectiveness of web-based physical–cognitive telerehabilitation exergaming on physical and cognitive performance in chronic stroke survivors. To our knowledge, this is the first RCT protocol to investigate the effectiveness of physical–cognitive training delivered via web-based telerehabilitation exergaming across a comprehensive range of domains, including motor function, gait speed, balance and limits of stability, fall risk, muscle power, and weight distribution, as well as global cognitive function, executive function, memory, and attention in individuals with chronic stroke. As research evidence has demonstrated the benefits of physical–cognitive training on health outcomes, it is proposed that web-based physical–cognitive telerehabilitation exergaming may further enhance both physical and cognitive functions beyond the effects of usual care. In addition to the accessibility and other advantages of digital technology, a home-based intervention delivered through web-based telerehabilitation exergaming with individualized training progression may improve adherence and provide a feasible and enjoyable intervention.

Given the long-term and chronic nature of stroke-related impairments, sustained engagement in physical activity and rehabilitation is essential to optimize and maintain long-term functional outcomes. However, access to rehabilitation services is often limited by geographical, financial, and logistical barriers, particularly for individuals living in the community after hospital discharge. Web-based telerehabilitation may address these challenges by enabling continuous, home-based training while reducing the burden associated with travel and service availability. Within this context, the present study protocol provides a rigorous framework for evaluating the clinical effectiveness of a web-based physical–cognitive telerehabilitation exergaming intervention. The findings from the planned RCT will contribute important evidence regarding the potential of digitally delivered, home-based rehabilitation to enhance physical and cognitive outcomes in chronic stroke survivors. If proven effective, this intervention may support the development of scalable and accessible rehabilitation models that complement usual care and inform future clinical practice and health service delivery. Remote delivery via telerehabilitation may bridge gaps in healthcare access caused by geographic distances. The novel web-based physical–cognitive telerehabilitation exergaming may contribute to clinical applications and promote therapeutic outcomes for chronic stroke survivors.

There are some limitations to this study. First, to control for potential spontaneous recovery effects, all participants will be recruited exclusively from the chronic stage of stroke. Therefore, the study findings may not be generalizable to individuals in the acute or subacute stages. Second, participation in the present study is limited to individuals who are regular users of a mobile phone, tablet, or notebook computer, which may introduce selection bias. In addition, this may also contribute to digital inequality, as not all patients have equal access to the internet or possess the necessary digital literacy to engage with the intervention. Lastly, as this study focuses on short- to medium-term outcomes following 12 weeks of training, the long-term sustainability of the intervention effects will require further investigation.

## Figures and Tables

**Table 1:** Schedule of enrolment, interventions, and assessments.

Timepoint	Study Period			
	Enrolment	Allocation	Post-Allocation	Follow-Up
	−t_1_	t_0_	0–12 Weeks	t_2_ 12 Weeks
Enrolment:				
Eligibility screen	X			
Informed consent	X			
Allocation		X		
Interventions:				
Intervention group			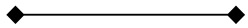	
Control group			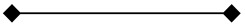	
Assessments and Measurements:				
TMT		X		X
TUG		X		X
10MWT		X		X
MoCA test		X		X
Digit Span Test		X		X
MAS		X		X
MFRT		X		X
5TSTS		X		X
SST		X		X
Body composition		X		X
PACES			X (after 1 week)	X

Note. TMT: Trail Making Test; TUG: Timed Up and Go test; 10MWT: 10-Meter Walk Test; MoCA: Montreal Cognitive Assessment; MAS: Motor Assessment Scale; MFRT: Modified Functional Reach Test; 5TSTS: 5 Times Sit-To-Stand test; SST: Stance Symmetry Test; PACES: Physical Activity Enjoyment Scale. X indicates that the assessment was performed at that timepoint.

## Data Availability

No new data were created or analyzed in this study.

## Supplementary Materials

The following supporting information can be downloaded at https://www.mdpi.com/article/10.3390/jcm15082945/s1, Supplementary File S1, Table S1: SPIRIT 2025 checklist of items to address in a randomized trial protocol. Supplementary File S2, Table S2: Detailed description of the web-based physical–cognitive telerehabilitation exergaming protocol and progression.

## Abbreviations

The following abbreviations are used in this manuscript:

TMT	Trail Making Test
TUG	Timed Up and Go test
10MWT	10-Meter Walk Test
MoCA	Montreal Cognitive Assessment
MAS	Motor Assessment Scale
MFRT	Modified Functional Reach Test
5TSTS	5 Times Sit-To-Stand test
SST	Stance Symmetry Test
PACES	Physical Activity Enjoyment Scale
