# Supplementary material for: Effects of Web-Based Physical–Cognitive Telerehabilitation Exergaming on Physical and Cognitive Performance in Chronic Stroke Survivors: Protocol for a Randomized Controlled Trial
Source: J Clin Med. 2026 Apr 13;15(8):2945. doi: 10.3390/jcm15082945 (PMC13116758; doi:10.3390/jcm15082945)
Supplement: Supplementary file 1 [file jcm-15-02945-s001.zip › jcm-4217768-supplementary- File S2.pdf]

Supplementary Table S2. Detailed Description of the Web-Based Physical–Cognitive Telerehabilitation Exergaming Protocol and Progression

Intervention	Descriptions and Game scenarios	Aims	Difficulty Level
**Physical training session (~45 minutes)**	• Participants will move their upper and lower extremities by following on-screen instructions delivered via the ExerbrainCMU platform.<br>• The exercises will include upper and lower trunk rotation, anteroposterior pelvic tilt, trunk inclination, performing D1 and D2 PNF patterns, alternating front punches, seated heel jacks, alternating taps, overhead reach, sit-to-stand exercises, multi-directional weight shifting, marching in both seated and standing positions, heel jacks, squats, and side-walking exercises.	• To enhance balance, aerobic capacity, motor performance in both the upper and lower extremities	• Level 1: 30 exercises, 5 repetitions per exercise, 2 sets, at an intensity of RPE 12–14, with normal movement complexity, normal speed, and simple directions<br>• Level 2: 30 exercises, 5 repetitions per exercise, 2 sets, at an intensity of RPE 12–14, with moderate movement complexity, moderate speed, and multi-directional movements<br>• Level 3: 30 exercises, 5 repetitions per exercise, 2 sets, at an intensity of RPE 15–17, with high movement complexity, faster speed, complex movements, and multi-directional movements

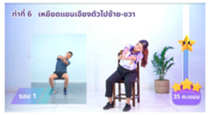

Seated exercises

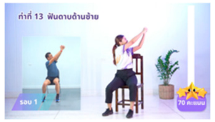

Performing D1-D2 PNF patterns

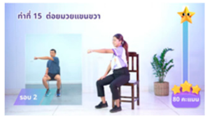

Alternating front punches

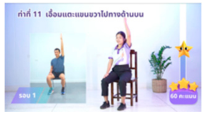

Overhead reach marching

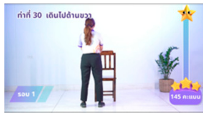

Side-walking exercises

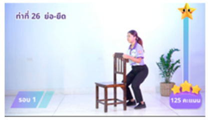

Squats

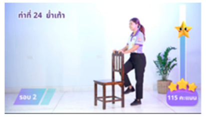

Marching

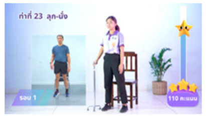

Sit-to-stand exercises

**Cognitive training session (~15 minutes)**	• Cognitive exercises will be delivered via the ExerbrainCMU platform according to the conditions of each game.<br><br>**Clock Game:**<br>Participants will be instructed to remember a sequence of numbers presented on the screen and then move their bodies according to the remembered sequence (e.g., 12 → 2 → 6)<br>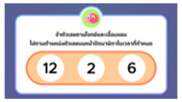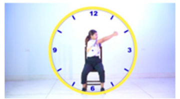	• To enhance memory, visuospatial ability, and dual-task performance	↑ Number of stimuli to be remembered *(Level 1: 3 numbers; Level 2: 4 numbers; Level 3: 5 numbers)*<br>↑ Attentional demand<br>↑ Cognitive complexity
	**Picture memory game:**<br>Participants will be instructed to memorize images (e.g., a notebook, a basketball, a bag, and glasses) presented on the screen. Afterward, participants will be required to select the recalled image from memory.<br>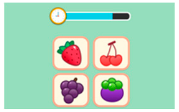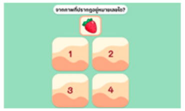	• To enhance memory and visuospatial ability	↑ Number of target images *(Level 1: 4 images; Level 2: 6 images; Level 3: 8 images)*<br>↑ Attentional demand<br>↑ Cognitive complexity<br>↓ Duration of memory retention *(Level 1: 20s; Level 2: 15s; Level 3: 10s)*
	**Bomb game:**<br>Animals and bombs will be presented on the screen. Participants will be instructed to tap on animals as quickly as possible while inhibiting responses to bombs.<br>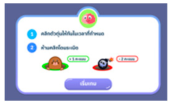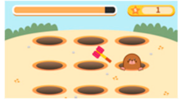	• To enhance attention and executive function	↑ Number of animals and bombs *(Level 1: 10 animals with 5 bombs; Level 2: 15 animals with 7 bombs; Level 3: 20 animals with 10 bombs)*<br>↑ Attentional demand<br>↑ Cognitive complexity

**Math game:** The task condition and numerical options will be presented on the screen in the form of bubbles. Participants will be instructed to perform the task according to the specified condition, such as arranging numbers in ascending or descending order, or selecting numbers based on their properties (e.g., even or odd numbers). 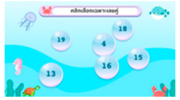 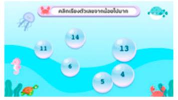	• To enhance attention and executive function	↑ Number of conditions and numerical options *(Level 1: 6 numbers; Level 2: 8 numbers; Level 3: 10 numbers)*<br>↑ Attentional demand<br>↑ Cognitive complexity
**Running game:** Participants will be required to solve arithmetic problems by selecting the correct answers while simultaneously alternating between corn collection and arithmetic task performance. 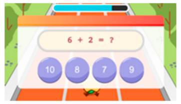 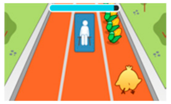	• To enhance attention, executive function, and dual-task performance	↑ Type of arithmetic tasks *(Level 1: single-digit arithmetic tasks; Level 2: two-digit arithmetic tasks; Level 3: three-digit arithmetic tasks)*<br>↑ Attentional demand<br>↑ Cognitive complexity
• **Visual matching:** Participants will memorize images based on specific features (e.g., size, shape, and color) and select the matching image from memory. 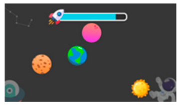 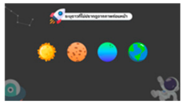	• To enhance attention, memory and visuospatial ability	↑ Number of images *(Level 1: 4 images; Level 2: 6 images; Level 3: 8 images)*<br>↑ Attentional demand<br>↑ Cognitive complexity<br>↓ Duration of memory retention *(Level 1: 20s; Level 2: 15s; Level 3: 10s)*

• **Pair matching:** Participants will memorize pairs of identical images along with their positions on the screen. Afterward, they will be instructed to match the previously memorized images. 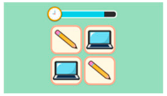 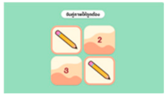	• To enhance attention, memory and visuospatial ability	↑ Number of images *(Level 1: 4 images; Level 2: 6 images; Level 3: 8 images)*<br>↑ Attentional demand<br>↑ Cognitive complexity<br>↓ Duration of memory retention *(Level 1: 20s; Level 2: 15s; Level 3: 10s)*
